# Uncovering survivorship bias in longitudinal mental health surveys during the COVID-19 pandemic

**DOI:** 10.1017/S204579602100038X

**Published:** 2021-05-26

**Authors:** Mark É. Czeisler, Joshua F. Wiley, Charles A. Czeisler, Shantha M.W. Rajaratnam, Mark E. Howard

**Affiliations:** 1 Turner Institute for Brain and Mental Health and School of Psychological Sciences, Monash University, Melbourne, Victoria, Australia; 2 Institute for Breathing and Sleep, Austin Health, Melbourne, Victoria, Australia; 3 Department of Psychiatry, Brigham & Women's Hospital, Boston, Massachusetts, USA; 4 Division of Sleep and Circadian Disorders, Departments of Medicine and Neurology, Brigham & Women's Hospital, Boston, Massachusetts, USA; 5 Division of Sleep Medicine, Harvard Medical School, Boston, Massachusetts, USA; 6 Division of Medicine, University of Melbourne, Melbourne, Victoria, Australia

**Keywords:** Epidemiology, non-random attrition, non-response bias, research design and methods

## Abstract

**Aims:**

Markedly elevated adverse mental health symptoms were widely observed early in the coronavirus disease-2019 (COVID-19) pandemic. Unlike the U.S., where cross-sectional data indicate anxiety and depression symptoms have remained elevated, such symptoms reportedly declined in the U.K., according to analysis of repeated measures from a large-scale longitudinal study. However, nearly 40% of U.K. respondents (those who did not complete multiple follow-up surveys) were excluded from analysis, suggesting that survivorship bias might partially explain this discrepancy. We therefore sought to assess survivorship bias among participants in our longitudinal survey study as part of The COVID-19 Outbreak Public Evaluation (COPE) Initiative.

**Methods:**

Survivorship bias was assessed in 4039 U.S. respondents who completed surveys including the assessment of mental health as part of The COPE Initiative in April 2020 and were invited to complete follow-up surveys. Participants completed validated screening instruments for symptoms of anxiety, depression and insomnia. Survivorship bias was assessed for (1) demographic differences in follow-up survey participation, (2) differences in initial adverse mental health symptom prevalence adjusted for demographic factors and (3) differences in follow-up survey participation based on mental health experiences adjusted for demographic factors.

**Results:**

Adjusting for demographics, individuals who completed only one or two out of four surveys had significantly higher prevalence of anxiety and depression symptoms in April 2020 (e.g. one-survey *v*. four-survey, anxiety symptoms, adjusted prevalence ratio [aPR]: 1.30, 95% confidence interval [CI]: 1.08–1.55, *p* = 0.0045; depression symptoms, aPR: 1.43, 95% CI: 1.17–1.75, *p* = 0.00052). Moreover, individuals who experienced incident anxiety or depression symptoms had significantly higher adjusted odds of not completing follow-up surveys (adjusted odds ratio [aOR]: 1.68, 95% CI: 1.22–2.31, *p* = 0.0015, aOR: 1.56, 95% CI: 1.15–2.12, *p* = 0.0046, respectively).

**Conclusions:**

Our findings reveal significant survivorship bias among longitudinal survey respondents, indicating that restricting analytic samples to only respondents who provide repeated assessments in longitudinal survey studies could lead to overly optimistic interpretations of mental health trends over time. Cross-sectional or planned missing data designs may provide more accurate estimates of population-level adverse mental health symptom prevalence than longitudinal surveys.

## Introduction

Studies have documented acutely elevated prevalence of adverse mental health symptoms during the early months of the coronavirus disease 2019 (COVID-19) pandemic compared with pre-pandemic data (CDC, 2020; Ettman *et al*., 2020; Li *et al*., 2020; Vindegaard and Benros, 2020; Wang *et al*., 2020; Pierce *et al*., 2020*a*; Bonati *et al*., 2021; Browning *et al*., 2021; Czeisler *et al*., 2021*b*). Prevalence of clinically significant mental distress rose by approximately 40% in the U.K. (Pierce *et al*., 2020*a*), and prevalence of anxiety and depression symptoms more than tripled in the United States (Czeisler *et al*., 2020; Ettman *et al*., 2020; Czeisler *et al*., 2021*b*). Analysis of longitudinal U.K. and U.S. survey data suggested that those increased prevalence may have been transient, with anxiety and depression symptoms declining among participants who completed several follow-up measures between March or April and August 2020 (Fancourt *et al*., 2020; Riehm *et al*., 2021). However, those longitudinal data from repeat-responders are not consistent with cross-sectional U.S. survey data, which indicate that levels of adverse mental health symptoms have remained persistently elevated (CDC, 2020; Vahratian *et al*., 2021; Czeisler *et al*., 2021*c*). As, for example, 38.5% of U.K. respondents were excluded from analysis because they did not complete multiple follow-up surveys, we analysed data from U.S. adults invited to complete surveys over a comparable time interval to determine if survivorship bias could account for the discrepancy between the published cross-sectional and longitudinal data from U.S. and U.K. This investigation has practical and theoretical implications. Reliable assessment of the prevalence of adverse mental health symptoms could both affect planning and resource allocation for mental health support services during the COVID-19 pandemic (Holmes *et al*., 2020), and inform policymakers of the mental health implications of issuing and lifting COVID-19 prevention measures of varying duration and intensity to balance against the transmission dynamics of severe acute respiratory coronavirus syndrome 2 (SARS-CoV-2) (Kissler *et al*., 2020; Batabyal, 2021; Batabyal and Batabyal, 2021). More broadly, given that survivorship bias has not previously been reported to affect large-scale internet-based mental health surveys, this investigation may influence mental health surveillance study design and interpretation of ongoing studies and previously published papers.

Survivorship bias occurs whenever missingness occurs by a non-random mechanism. Therefore, while bias induced by demographic differences in follow-up survey participation may be reduced by poststratification weighting for observed variables using population estimates (Corry *et al*., 2017), this strategy cannot account for survivorship bias. Survivorship bias can be problematic if individuals who make it past a selection process are different than those who do not. In the context of longitudinal mental health surveys, bias introduced by non-random differences in baseline mental health or mental health trajectories could result from restricting an analytic sample to respondents who consistently participated in surveys, ignoring individuals who dropped out. If the people who dropped out (i.e. study non-survivors) were to have meaningfully different baseline mental health or mental health trajectories than those who remain active study participants (i.e. study survivors), the resulting analytic sample would be non-representative.

Longitudinal studies have provided evidence of survivorship bias related to mental health within specific populations (Herbert *et al*., 1992; Neuner *et al*., 2007; Kakudate *et al*., 2010; Lamers *et al*., 2012; de Graaf *et al*., 2013; Mayeda *et al*., 2018; Ramsey *et al*., 2019; Kigawa *et al*., 2019*a*, *b*; Cornish *et al*., 2021). For example, diagnosed depression has been associated with lower participation in follow-up surveys in parents and children (Mayeda *et al*., 2018; Cornish *et al*., 2021) and a naturalistic cohort on depression and anxiety (Lamers *et al*., 2012), while assessment of three-year follow-up surveys in the Netherlands general population reported no association between mental health status at baseline and attrition (de Graaf *et al*., 2013). However, considerable effort was exerted by de Graaf *et al.* to optimise participation, including a two-year initial contact and follow-up intervals, multiple attempts to recontact participants and frequent contact between interviews. Other studies have found that cancer survivors who completed surveys at multiple time points had higher health-related quality of life scores than those who completed surveys at a single timepoint (Ramsey *et al*., 2019) and pregnant persons with psychological distress had higher odds of not completing follow-up surveys compared with pregnant persons without such distress (Kigawa *et al*., 2019*b*). Additionally, non-participation in follow-up surveys has been associated with smoking and alcohol use among trauma patients (Neuner *et al*., 2007), and with lower perceived oral healthcare-specific self-efficacy among patients with chronic periodontitis (Kakudate *et al*., 2010). Finally, of 294 women who presented at an emergency department following sexual assault, 136 (46%) could not be reached within 48 h and 233 (79%) did not participate in six-month follow-up (Herbert *et al*., 1992). While anxiety and depression symptom ratings were attenuated in the analytic sample of 61 women who completed six-month follow-up surveys, women with higher rape-trauma-symptom scores were more likely to decline follow-up surveys. If survivorship bias existed in that study, generalising data supporting declining adverse mental health levels from only those with lower initial rape-trauma-symptom scores could lead to an overly optimistic interpretation of mental health following sexual assault.

To our knowledge, survivorship bias assessment has not been described and is seldom addressed in longitudinal mental health internet-based survey data collected from the general population. As numerous studies have responded to the call for mental health research by launching longitudinal mental health survey studies, we undertook a robust assessment of potential survivorship bias in our longitudinal mental health survey study.

## Methods

### Study design

We conducted a retrospective analysis of U.S. participants in The COVID-19 Outbreak Public Evaluation (COPE) Initiative (www.thecopeinitiative.org) (Czeisler *et al*., 2021*a*). Internet-based surveys were administered through Qualtrics, LLC (Qualtrics, 2020) to 4042 U.S. adults aged ⩾18 years during 2–8 April 2020 (April-2020). For the April-2020 wave, demographic quota sampling for gender, age, race and ethnicity was employed to recruit respondents such that each cross-sectional sample matched 2010 U.S. Census national population estimates for these characteristics. The sample included 3010 (74.5%) from across the U.S., plus additional respondents from New York City (*n*: 507 [12.5%]) and Los Angeles (*n*: 525 [13.0%]) to recruit participants from cities with different prevalence of SARS-CoV-2 during the early months of the pandemic (Czeisler *et al*., 2021*b*). All respondents were invited to complete follow-up surveys during 5–12 May 2020 (May-2020) and 24–30 June 2020 (June-2020). Respondents who completed at least one of these follow-up surveys were also invited to complete surveys during 28 August to 6 September 2020 (September-2020). To account for any deviations from the April-2020 demographic recruitment quotas, survey weighting (iterative proportional fitting) was employed to match improved sample representativeness by gender, age and combined race/ethnicity using Census population estimates. Given the bias-variance compromises associated with trimming survey weights (Lee *et al*., 2011), no trimming was conducted on the primary analytic sample, which had minimum and maximum weights of 0.71 and 1.80, respectively. As gender data were not available in the 2010 U.S. Census, for this analysis, sex was used for weighting of dichotomised gender. One respondent who was inadvertently invited to and completed a September-2020 survey after not having participated in May-2020 or June-2020 surveys, and two respondents who identified as ‘Other’ gender, were not included in this analysis.

Surveys contained demographic questions and assessed public attitudes and behaviours related to the pandemic and its mitigation, along with mental health symptoms. Validated screening instruments and modified questions from instruments were used. Among the adverse mental health symptom screening instruments administered were the 4-item Patient Health Questionnaire (PHQ-4) (Löwe *et al*., 2004, 2010), with subscales for assessment of anxiety (2-item Generalised Anxiety Disorder [GAD-2]) and depression (2-item PHQ [PHQ-2]) symptoms, and the 2-item Sleep Condition Indicator (SCI-02) for assessment of insomnia symptoms (Espie *et al*., 2014).

### Statistical analysis

We explored whether potential mental health survivorship bias could be explained by: (1) demographic differences in repeated-measures respondents (i.e. cross-sectional *v*. longitudinal respondents differing in their demographics, but mental health being similar among members of a demographic subgroup); or (2) differences being within demographic subgroups. Demographic survey weighting could considerably reduce bias in the first, but not second scenario.

Potential demographic differences in survey retention were assessed using Chi-square tests with design effect correction factors (Walker and Young, 2003) to assess for differences between the percentages of respondents who completed one, two, three or four surveys by gender, age group in years, combined race/ethnicity, education attainment and 2019 household income. Potential differences in baseline mental health measures were assessed using weighted Poisson regression models with robust standard error estimators to estimate prevalence ratios (PRs) and 95% confidence intervals (CIs) for April-2020 anxiety symptoms (⩾3 out of 6 on the GAD-2 subscale of the PHQ-4), depression symptoms (⩾3 out of 6 on the PHQ-2 subscale of the PHQ-4), and insomnia symptoms (⩽2 out of 8 on the SCI-02). With the reference group as four-survey respondents (i.e. the group that would be included in a longitudinal analytic sample that excluded non-responders), PRs and aPRs were estimated for one-survey, two-survey and three-survey respondents. Adjusted Poisson regression models included gender, age group, race/ethnicity, education attainment and 2019 household income as covariates. Next, to assess for potential differences in population estimates for the prevalence of anxiety, depression and insomnia symptoms in April 2020 using samples with differing retention over time, the April-2020 sample was separated into four groups: respondents who completed one, two, three or four surveys through September 2020. Each group was separately weighted to match national U.S. population estimates by gender, age and race/ethnicity, with survey weights trimmed between 1/3 and 3 to account for otherwise-extreme weights due to demographic differences in survey completion rate (e.g. sample of respondents who completed four surveys, maximum weight before trimming: 17.24). Prevalence estimates for anxiety, depression and insomnia symptoms were made for each possible grouping (number of completed surveys, one *v*. two, one *v*. three, one *v*. four, two *v*. three, two *v*. four and three *v*. four) based on these demographically representative groups. Chi-square tests with design effect correction factors were used to assess for different point estimates for prevalence of April-2020 anxiety, depression and insomnia symptoms between groups.

To evaluate potential differences in trajectories of adverse mental health symptoms over time by number of completed surveys, prevalence of symptoms of anxiety, depression and insomnia over two timepoints (April-2020 to May-2020 and April-2020 to June-2020) among respondents who completed all four surveys was compared with the prevalence among those who completed two total surveys (only April-2020 and May-2020 or only April-2020 and June-2020, which are the only two possible groupings of two-survey respondents, as April-2020 respondents who did not complete surveys in May-2020 or June-2020 were not invited to complete September-2020 surveys). Respondents who participated in all four surveys completed three of three follow-up surveys (100% retention rate), whereas respondents who participated in two surveys only completed one of three follow-up surveys (33% retention rate). Chi-square tests with design effect correction factors were used to assess for differences in initial (April-2020) prevalence between samples, and McNemar's Chi-square tests were used to test for differences over time among paired data within each sample (e.g. April-2020 *v*. May-2020 and April-2020 *v*. June-2020 among respondents who completed these surveys sequentially). Prevalence ratios were used to estimate differences in prevalence between subsamples over time.

Finally, to assess whether changes in mental health symptoms were associated with differential participation in follow-up surveys, weighted ordinal logistic regressions were used to estimate odds ratios for lower participation in June-2020 and September-2020 surveys among respondents who completed April-2020 and May-2020 surveys based on symptoms of anxiety, depression or insomnia reported in these two initial surveys. For each of these adverse mental health conditions over April-2020 and May-2020, respondents were categorised as having no symptoms at either timepoint (Neither), symptoms at both timepoints (Both), incident symptoms in May-2020 after not having experienced symptoms in April-2020 (Incidence), or remitted symptoms in May-2020 after having experienced symptoms in April-2020 (Remission). Odds ratios for lower participation in follow-up surveys were estimated with the dependent variables ordered as 0 (completed both follow-up surveys), 1 (completed one follow-up survey [either June-2020 or September-2020]), and 2 (completed neither follow-up survey). Odds were estimated both unadjusted and adjusted for gender, age group, race/ethnicity, education attainment and 2019 household income. Statistical significance was determined at *α*: 0.025 to account for multiple comparisons. The proportionality assumption of the outcomes in the ordinal logistic regression models was assessed using the Brant test (Brant, 1990), which indicated that the proportional odds assumption held for the Omnibus test for all models.

### Study approval and informed consent

The Monash University Human Research Ethics Committee approved the study protocol. Participants provided electronic informed consent. Rounded weighted values are reported unless otherwise specified. Analyses were conducted in R (version 4.0.2; The R Foundation) with the R survey package (version 3.29) and Python (version 3.7.8).

## Results

Overall, 4042 of 6548 (61.7%) eligible invited adults completed surveys during the first wave of The COVID-19 Outbreak Public Evaluation (COPE) Initiative, administered during 2–8 April 2020. Of 4039 (99.9%) who provided answers to questions used for survey weighting and were therefore included in this analysis, 2098 (51.9%) completed May-2020 surveys, 1619 (40.1%) completed June-2020 surveys, and 1151 (28.5%) completed September-2020 surveys. In total, 1712 (42.4%) completed one survey, 725 (17.9%) completed two surveys, 663 (16.4%) completed three surveys, and 939 (23.2%) completed all four surveys (Table 1). By age, 76.0% of respondents aged 18–24 years completed one survey, whereas 7.3% completed three or four surveys. In contrast, just 12.1% of respondents aged ⩾65 years completed one survey, compared with 72.5% who completed three of four surveys (*p* < 2.20 × 10^−16^). By race/ethnicity, non-Hispanic White and non-Hispanic Asian respondents had the lowest prevalence of one-survey respondents (33.4% and 32.3%, respectively) and highest prevalence of four-survey respondents (29.7% and 23.9%), whereas non-Hispanic Black and Latinx respondents had the highest prevalence of one-survey respondents (65.7% and 60.7%, respectively) and lowest prevalence of four-survey respondents (8.6% and 10.1%); *p* < 2.20 × 10^−16^. Percentage of completed surveys also increased significantly with higher education attainment (e.g. one-survey, high school diploma or less: 52.9%, after bachelor's degree: 33.1%, *p* < 2.20 × 10^−16^) and higher 2019 household income (e.g. one-survey, USD <25 000: 51.0%, ⩾100 000: 37.0%, *p* = 1.56 × 10^−9^).

**Table 1.** Respondent characteristics, overall and by number of completed surveys

	Number of respondents				Number of completed surveys								
	Unweighted		Weighted		One		Two		Three		Four		Chi-Sq
	*n*	(%)	*n*	(%)	*n*	(%)	*n*	(%)	*n*	(%)	*n*	(%)	*p*^a^
Total	4039	(100)	4039	(100)	1712	(42.4)	725	(17.9)	663	(16.4)	939	(23.2)	–
Gender													
Male	1814	(44.9)	1986	(49.2)	872	(43.9)	329	(16.6)	307	(15.5)	477	(24.0)	0.032
Female	2225	(55.1)	2053	(50.8)	840	(40.9)	395	(19.2)	356	(17.3)	462	(22.5)	
Age group in years													
18–24	456	(11.3)	528	(13.1)	401	(76.0)	88	(16.7)	22	(4.2)	17	(3.1)	<2.20 × 10^−16^^b^
25–44	1335	(33.1)	1414	(35.0)	809	(57.2)	269	(19.0)	182	(12.8)	155	(11.0)	
45–64	1420	(35.2)	1403	(34.7)	418	(29.8)	261	(18.6)	291	(20.8)	433	(30.8)	
⩾65	828	(20.5)	693	(17.2)	84	(12.1)	107	(15.4)	168	(24.2)	335	(48.3)	
Race/ethnicity^c^													
White, non-Hispanic	2937	(72.7)	2575	(63.7)	860	(33.4)	459	(17.8)	491	(19.1)	765	(29.7)	<2.20 × 10^−16^^b^
Black, non-Hispanic	329	(8.1)	493	(12.2)	324	(65.7)	72	(14.6)	55	(11.1)	43	(8.6)	
Asian, non-Hispanic	224	(5.5)	189	(4.7)	61	(32.3)	45	(24.0)	37	(19.8)	45	(23.9)	
Other, non-Hispanic	126	(3.1)	122	(3.0)	66	(54.4)	22	(18.5)	13	(11.0)	20	(16.1)	
Latinx, any race or races	423	(10.5)	660	(16.3)	401	(60.7)	126	(19.0)	67	(10.1)	67	(10.1)	
Education attainment^d^													
⩽ High school diploma	735	(18.2)	777	(19.2)	411	(52.9)	137	(17.7)	86	(11.0)	143	(18.4)	<2.20 × 10^−16^^b^
College or some college	2484	(61.5)	2473	(61.2)	1023	(41.4)	445	(18.0)	426	(17.2)	579	(23.4)	
> Bachelor's degree	792	(19.6)	756	(18.7)	250	(33.1)	142	(18.8)	150	(19.8)	215	(28.4)	
2019 Household income (USD)													
< 25 000	578	(14.3)	607	(15.0)	309	(51.0)	111	(18.3)	86	(14.1)	101	(16.7)	1.56 × 10^−9^^b^
25 000–49 999	816	(20.2)	834	(20.6)	395	(47.4)	155	(18.5)	112	(13.5)	172	(20.6)	
50 000–99 999	1291	(32.0)	1271	(31.5)	489	(38.5)	227	(17.9)	235	(18.5)	320	(25.1)	
⩾100 000	1156	(28.6)	1125	(27.9)	416	(37.0)	202	(17.9)	203	(18.0)	305	(27.1)	
Unknown	198	(4.9)	202	(5.0)	103	(51.0)	31	(15.1)	27	(13.3)	42	(20.6)	

USD = United States Dollars.

a Chi-square *p* value across all groups within a demographic subgroup (e.g. across all age groups). Chi-square tests included design effect correction factors.

b *p* < 0.025.

c The ‘Other, non-Hispanic,’ category includes respondents who identified as not Hispanic or Latino and as more than one race or as American Indian or Alaska Native, Native Hawaiian or Pacific Islander, or Other.

d The response option ‘Unknown’ is not shown due to small counts (*n* = 34 total).

**Fig. 1.**
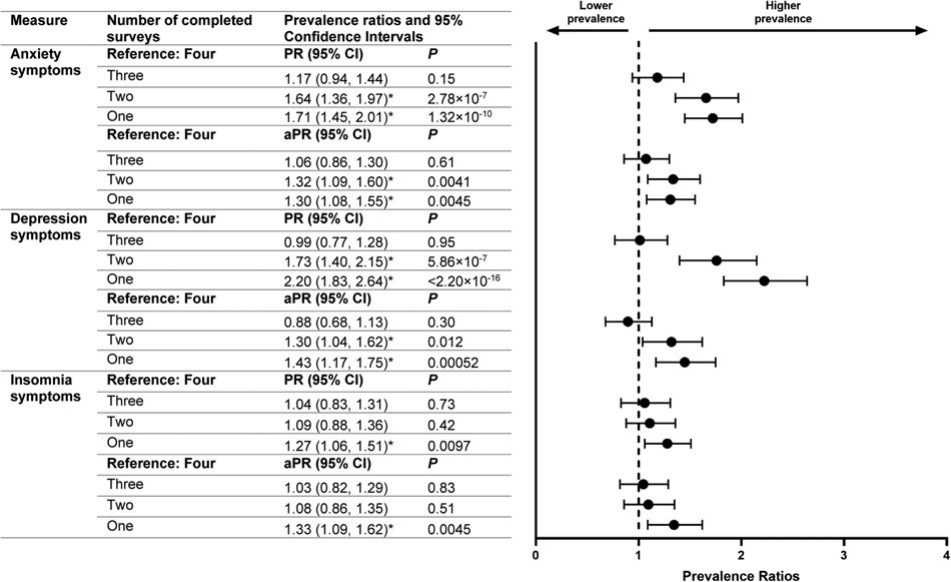
Crude and adjusted prevalence ratios for anxiety, depression and insomnia symptoms in April 2020 by number of completed surveys. The marker * indicates that *p* < 0.025 (i.e. the prevalence ratio is statistically significant).

**Fig. 2.**
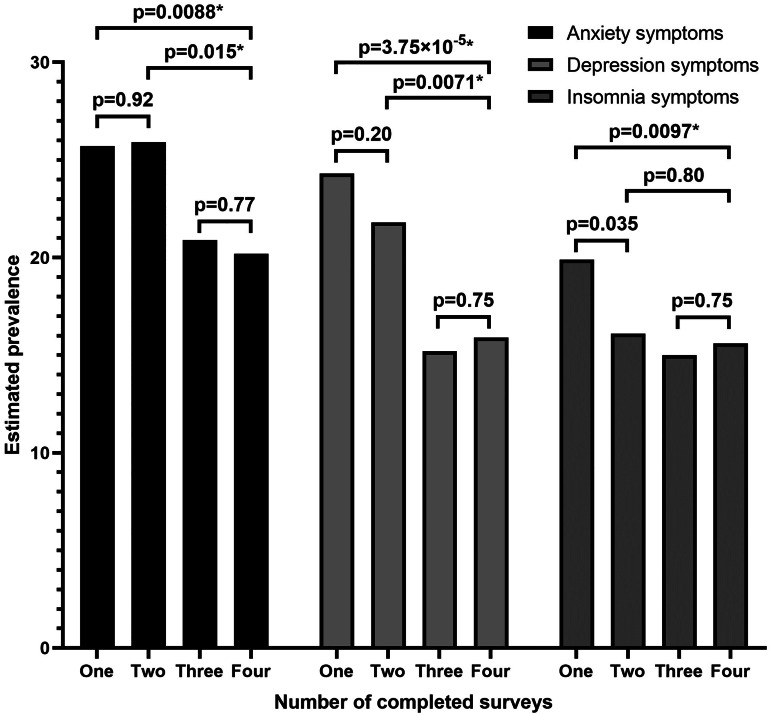
Estimated prevalence of symptoms of anxiety, depression and insomnia in April 2020 based on total number of completes surveys, with each group weighted to population estimates for gender, age and race/ethnicity. The marker * indicates that *p* < 0.025 (i.e. the difference in prevalence estimates is statistically significant). The rounded, weighted percentages of respondents shown in Fig. 2. based on the number of completed surveys may differ from those reported in Table 1 due to different survey weight raking and trimming.

Compared with respondents who completed all four surveys, those who completed only one or two surveys had higher prevalence of anxiety and depression symptoms in April-2020 surveys (Fig. 1). Differences remained after adjusting for gender, age, race/ethnicity, education attainment and 2019 household income among respondents (e.g. one-survey *v*. four-survey, anxiety symptoms, aPR: 1.30, 95% CI: 1.08–1.55, *p* = 0.0045; depression symptoms, 1.43, 1.17–1.75, *p* = 0.00052). Adjusted prevalence of insomnia symptoms in April-2020 was higher among individuals who completed only one survey compared with those who completed all four surveys (aPR: 1.33, 95% CI: 1.09–1.62, *p* = 0.0045). Prevalence estimates for April-2020 adverse mental health symptoms among groups of respondents who completed one, two, three or four surveys—each separately weighted to improve group representativeness of the U.S. population by gender, age and race/ethnicity—revealed that estimates for anxiety, depression and insomnia symptoms based on respondents who completed only one survey were higher than those for respondents who completed three or four surveys (e.g. one-survey *v*. four-survey, anxiety symptoms: 25.7% *v*. 20.2%, *p* = 0.088; depression symptoms: 24.3% *v*. 15.9%, *p* = 2.84 × 10^−5^; insomnia symptoms: 19.9% *v*. 15.6%, *p* = 0.022) (Fig. 2). Prevalence estimates for these symptoms were similar between one- and two-survey respondents, and between three- and four-survey respondents. Estimates for depression symptoms were also greater among respondents who completed two surveys compared with those who completed three or four surveys, while estimates for anxiety symptoms were greater among respondents who completed two surveys compared with those who completed four surveys.

**Fig. 3.**
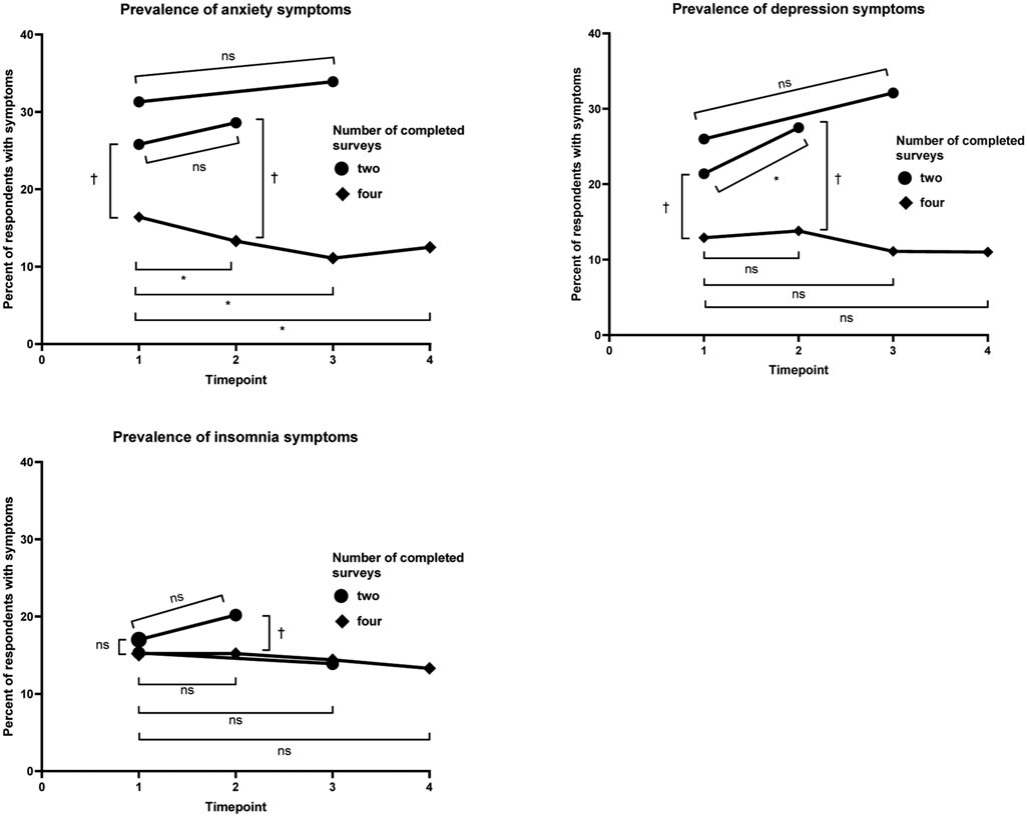
Longitudinal comparisons of anxiety and depression symptom prevalence by number of repeated measures.The marker * indicates that *p* < 0.025 within the same group over the timepoints designated with brackets (i.e. the prevalence estimates differ with statistical significance). The marker † indicates that *p* < 0.025 between groups at a single timepoint, with the comparison designated with brackets (i.e. the prevalence estimates differ with statistical significance). The marker ns indicates that *p* ≥ 0.025 (i.e. the prevalence estimates do not differ with statistical significance).

In the comparison of adverse mental health symptom prevalence among respondents who completed only two surveys *v*. those who completed all four surveys (*n*: 939), both two-survey groups (April-2020 and May-2020 only [April-and-May; *n*: 584], April-2020 and June-2020 only [April-and-June; *n*: 141]) started with higher April-2020 prevalence of anxiety and depression symptoms (April-and-May, anxiety symptoms PR: 1.57, depression symptoms PR: 1.66; April-and-June: 1.91 and 2.02, respectively), and the prevalence ratios increased for the second completed surveys (April-and-May: 2.15 and 1.99, respectively; April-and-June: 2.55 and 2.33, respectively) (Fig. 3). The prevalence of anxiety symptoms among April-and-May and April-and-June two-survey respondents was similar between surveys (April-and-May: 25.8% and 28.6%, respectively, *p* = 0.19; April-and-June: 31.3% and 33.9%, respectively, *p* = 0.57), whereas the prevalence of anxiety symptoms in four-survey respondents decreased over these intervals (April-and-May: 16.4% and 13.3%, *p* = 0.012; April-and-June: 16.4% and 11.1%, *p* = 1.11 × 10^−5^). The prevalence of depression symptoms increased among April-and-May two-survey respondents (21.4% and 27.5%, respectively, *p* = 0.0017), but not among four-survey respondents (12.9% and 13.8%, *p* = 0.45).

**Fig. 4.**
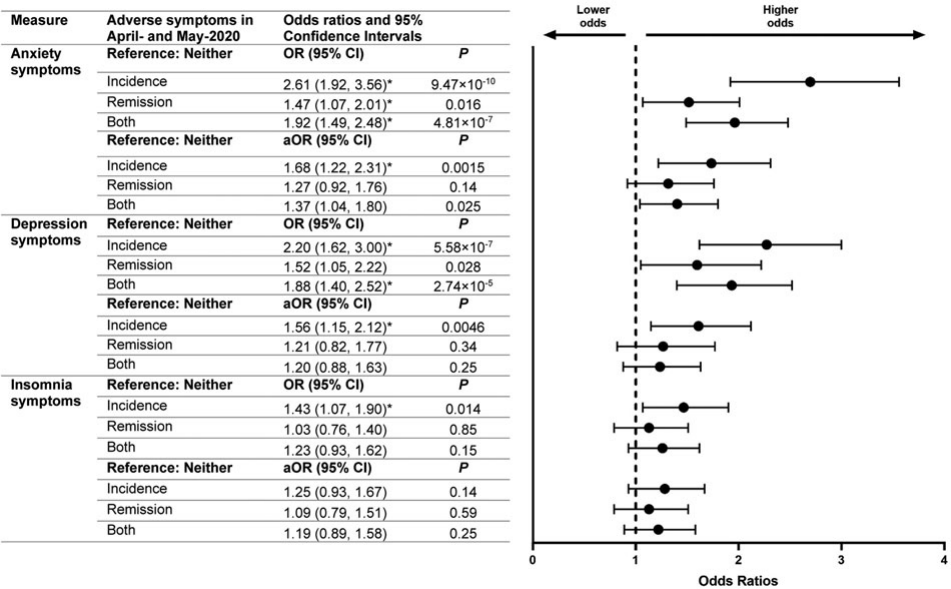
Odds of lower participation in follow-up surveys based on mental health in earlier surveys. The marker * indicates that *p* < 0.025 (i.e. the odds ratio is statistically significant).

Analysis of respondents who completed April-2020 and May-2020 surveys revealed that, compared with individuals who did not experience anxiety or depression symptoms during these initial surveys, those who experienced incident anxiety or depression symptoms had increased odds of lower participation in future follow-up surveys (i.e. June-2020 and September-2020) (Fig. 4). Individuals who experienced anxiety symptoms and depression symptoms in May-2020 after not having done so in April-2020 had 1.68-times (1.22–2.31, *p* = 0.0015) and 1.56-times (1.15–2.12, *p* = 0.0046) increased adjusted odds, respectively, of lower participation in June-2020 and September-2020 surveys. Adjusted odds of follow-up survey participation did not differ on the basis of insomnia symptoms, or among those who experienced: (1) remission of anxiety or depression symptoms or (2) persistent depression symptoms compared with those who did not experience these symptoms in April-2020 or May-2020. Individuals who experienced persistent anxiety symptoms, on the other hand, did have higher adjusted odds of lower participation in subsequent surveys (1.37, 1.04–1.80, *p* = 0.025). Though the magnitude of the adjusted odds ratios were higher for individuals with incident *v*. persistent adverse mental health symptoms, those who experienced incident symptoms did not have significantly higher adjusted odds of loss to follow-up compared to individuals who experienced persistent symptoms.

## Discussion

Analysis of mental health among survey respondents based on their participation in follow-up surveys revealed considerable survivorship bias related to: (1) demographic differences in survey retention; (2) differences in initial mental health, adjusted for gender, age, race/ethnicity, education and income and (3) higher odds of lower participation in follow-up surveys among respondents who experienced worsened mental health over time. The first of these forms of survivorship bias can be reduced by the application of poststratification weights. The second of these forms of survivorship bias precludes use of a longitudinal sample alone to estimate population prevalence of adverse mental health symptoms. However, simultaneous collection of cross-sectional data from representative samples of independent participants could inform strategies to mitigate differences in initial prevalence of adverse mental health symptoms, which could include adjustment for baseline differences in mental health between cross-sectional *v*. longitudinal respondents. The third of these forms of survivorship bias is most challenging to take into account given the unknown trajectories of respondents who do not consistently participate in follow-up surveys. Recognition that individuals who experienced incident anxiety or depression symptoms had higher odds of not completing follow-up surveys reveals the hazard of overlooking this form of survivorship bias, and should temper conclusions about trends of anxiety and depression symptoms in longitudinal mental health survey respondents, especially as generalising from repeated survey administration among longitudinal respondents without addressing these biases could lead to potentially erroneous conclusions (e.g. that adverse mental health symptom prevalence in a population are improving over time).

Understanding strengths and limitations of study approaches should inform the design and interpretation of findings (Pierce *et al*., 2020*b*). Longitudinal studies have advantages, including increased power to detect causal pathways and mediating factors, reduced reliance on recall bias, and establishment of the order in which events and outcomes occur. However, survivorship bias in longitudinal mental health surveys suggest that longitudinal samples may be non-representative of population-level mental health. While unable to determine causation, cross-sectional studies can more rapidly generate data, and our data provide further evidence that cross-sectional data may be more reliable for the assessment of population-level prevalence of adverse mental health symptoms at a given timepoint (Sedgwick, 2014). Future study designs could include planned missing data designs (Rioux *et al*., 2020) to benefit from the strengths of these study designs while minimising associated biases. Researchers could explore different designs involving planned missingness in longitudinal mental health surveys, such as multiform (i.e. random assignment of participants to have missing questionnaire items), wave-missing (planned occasions of participants missing measurements), and two-method designs (using gold-standard methods on a random subset of respondents [e.g. clinical diagnosis of mental health conditions] of a large sample) (Rioux *et al*., 2020). Such designs are of heightened importance for cohort studies investigating neuropsychiatric symptoms and conditions among the myriad post-acute sequelae of COVID-19 (PASC) (Speth *et al*., 2020; Boldrini *et al*., 2021; Nalbandian *et al*., 2021; Perlis *et al*., 2021; Taquet *et al*., 2021*a*, *2021b*), as non-random loss to follow-up could influence estimates for incidence and presentations of PASC.

Strengths of this analysis include four timepoints to assess response bias, high initial response (61.7%) and retention (39.6% of respondents completed at least three of four surveys) rates, utilisation of clinically validated screening instruments, and implementation of quota sampling and survey weighting to improve sample representativeness by national estimates for gender, age and race/ethnicity. Moreover, multiple types of survivorship bias were assessed, including differential demographic attrition and demographic-adjusted assessment of both initial mental health as well as odds of participation in follow-up surveys based on changes to mental health over the initial two surveys. Finally, bias was assessed both cross-sectionally and longitudinally. The findings in this report are also subject to limitations. First, while this analysis focused on survivorship bias, these data may be subject to other biases, including recall and response biases (Infante-Rivard and Cusson, 2018; Adams *et al*., 2020); however, quota sampling and survey weighting were employed to reduce demographic-related response bias. Second, though strategies were used to improve sample representativeness, and this Internet-based survey sample should represent the adult U.S. population by gender, age and race/ethnicity, it may not fully represent all U.S. adults, especially with regards to Internet access. Third, April-2020 respondents who did not respond to invitations to complete surveys in either May-2020 or June-2020 were not invited to complete September-2020 surveys, so these respondents did not have the opportunity to complete September-2020 surveys. However, after having declined two successive invitations, it is unlikely that a substantial number of these respondents would have completed September-2020 surveys. Finally, portions of the sample were oversampled from the New York City and Los Angeles metropolitan areas. However, all 50 states and Washington D.C. were represented, and this analysis was not designed to produce national population estimates for adverse mental health symptoms. Nevertheless, sensitivity analyses were conducted for all regression models on the subset of 3008 nationwide respondents (i.e. excluding respondents intentionally recruited from the N.Y.C. and L.A. metropolitan areas). The magnitude and significance of associations between survey completion and adverse mental health symptoms were largely maintained, indicating that the inclusion of oversampled N.Y.C. and L.A. respondents did not systematically bias the findings.

Longitudinal survey-based assessment of mental health is a useful and widely used research method that can provide important insights gained from monitoring the same participants over time. However, our data demonstrate that analysing mental health trends among only individuals who consistently respond to longitudinal mental health surveys can lead to overly optimistic interpretations of mental health trends by excluding individuals who less frequently respond to follow-up survey invitations. Survivorship bias assessment should therefore be among bias assessments (Sanderson *et al*., 2007; Mayeda *et al*., 2016; Griffith *et al*., 2020; Czeisler *et al*., 2021*d*) applied before conclusions based on repeated assessments from participants in a longitudinal study are generalised, and decisions regarding the allocation of mental health resources should be informed by studies with measures to reduce these various biases. These data have critical implications for the design of future studies and interpretation of data from published papers and ongoing surveillance studies with longitudinal study designs, both during and beyond the COVID-19 pandemic.

## Data

All relevant data supporting the findings in this study are available from the corresponding author upon reasonable request. Reuse is permitted only following a written agreement from the corresponding author and primary Institution.
